# Digital Rectal Examination Is a Valuable Bedside Tool for Detecting Dyssynergic Defecation: A Diagnostic Study and a Meta-Analysis

**DOI:** 10.1155/2021/5685610

**Published:** 2021-10-28

**Authors:** Jie Liu, Chaolan Lv, Yizhou Huang, Ying Wang, Dandan Wu, Cong Zhang, Chenyu Sun, Wei Wang, Yue Yu

**Affiliations:** ^1^Department of Gastroenterology, The First Affiliated Hospital of USTC, Division of Life Sciences and Medicine, University of Science and Technology of China, No. 17 Lu Jiang Road, Hefei 230001, Anhui, China; ^2^Graduate School of Bengbu Medical College, Bengbu 233000, Anhui Province, China; ^3^Endoscopy Center Department, The First Affiliated Hospital of USTC, Division of Life Sciences and Medicine, University of Science and Technology of China, Hefei 230036, Anhui Province, China; ^4^AMITA Health Saint Joseph Hospital Chicago, 2900 N. Lake Shore Drive, Chicago 60657, IL, USA

## Abstract

**Background:**

Accurate dyssynergic defecation (DD) diagnosis depends on anorectal physiological tests that are not widely available.

**Aim:**

The purpose of this study is to evaluate the diagnostic yield of digital rectal examination (DRE) compared with anorectal physiological tests in diagnosing DD in patients with constipation.

**Methods:**

A total of 218 chronic constipation patients who fulfilled the Rome IV diagnostic criteria for functional constipation (FC) and underwent a standardized DRE and high-resolution anorectal manometry (HRAM) test were enrolled in this study. The diagnostic accuracy of DRE compared with HRAM was evaluated, and the agreement between DRE and HRAM was calculated. Furthermore, a comprehensive literature search on PubMed, Web of Science, Cochrane Library, and Embase database was conducted to further elucidate the pooled diagnostic accuracy of DRE in DD patients.

**Results:**

A total of 101 patients (46.33%) had a DD pattern using HRAM, while 117 patients (53.67%) were diagnosed without DD. The sensitivity of DRE in diagnosing dyssynergia was 71.3%, and the specificity was 76.1%. There was a moderate agreement between DRE and HRAM for diagnosing DD (*κ*-coefficient = 0.474, *P* < 0.001). Meanwhile, six studies (including our study) comprising 964 constipated patients were included in our meta-analysis. The outcomes demonstrated that the AUC was 0.85 (95% CI 0.82–0.88) with 77% summary sensitivity (95% CI 65–86) and 80% summary specificity (95% CI 71–86) to diagnose DD.

**Conclusions:**

DRE could be a valuable tool for screening DD. Our study revealed acceptable sensitivity and specificity of DRE in detecting dyssynergia compared with the physiological tests. Meanwhile, our study highlights that DRE remains an important tool in clinical practice.

## 1. Introduction

Dyssynergic defecation (DD) is an anorectal dysfunction characterized by impaired relaxation or improper contraction of pelvic floor muscles that occurs during attempted defecation [1] and has been implicated in persistent constipation. [2] According to previous studies, up to 17% of the global population suffer from chronic constipation, while more than 50% of patients referred to tertiary centers for chronic constipation treatment are diagnosed with DD [3, 4]. In addition to Rome IV diagnostic criteria, diagnosing DD depends on physiological tests, including evidence of abnormal coordination pattern of defecation detected by anorectal manometry and/or electromyographic. Moreover, DD diagnosis is also supported by prolonged balloon expulsion time, abnormal retention of radiopaque markers, and inability to evacuate barium from the rectum and physical examination [1, 5]. Notably, initial physical examination, particularly a thorough digital rectal examination (DRE), is critical in evaluating constipated patients [6].

Physiological tests are critical in DD diagnosis and are considered the most important standard for DD [1]. DRE has been confirmed to be a reliable tool for detecting DD and facilitating selection of patients for additional physiological tests [7, 8]. DRE can identify the presence of structural abnormalities, such as a stricture or spasm, tenderness, mass, or stool blockage [9]. Generally, the resting and squeeze anal sphincter tones are within a particular range. When individuals were requested to push and bear down as if to defecate with a normal push maneuvre, the examiner could feel that external anal sphincter and/or puborectalis muscle of subjects were relaxed, together with perineal descent and tightening of abdominal muscles. However, one or more of these responses are absent in DD patients [7, 10].

The accurate DD diagnosis depends on anorectal physiological tests that are not readily available. DRE is a simple and inexpensive tool widely available for diagnosing anorectal disorders in clinics. However, there is a lack of emphasis on DRE use. Lawrentschuk et al. [11] reported that only 11% of students had palpated constipated patients, and up to 17% did not perform it during medical school. In other words, it is either not performed or inadequately used in clinical practice in evaluating patients with functional anorectal disorders. The purpose of this study is to evaluate the diagnostic yield of DRE compared with high-resolution anorectal manometry (HRAM) in diagnosing DD in patients with functional constipation. To further elucidate the pooled diagnostic accuracy of DRE in DD patients, we conducted a meta-analysis using data from previously published research, including our study.

## 2. Diagnostic Study

### 2.1. Materials and Methods

#### 2.1.1. Study Population

This study recruited constipated patients (outpatients and inpatients) who fulfilled the Rome IV diagnostic criteria [12] for functional constipation (FC) at the Gastrointestinal Motility Center, the First Affiliated Hospital of University of Science and Technology of China, from November 2016 to January 2021. The data were analyzed prospectively from registered patients at our motility center. The inclusion criteria were as follows: FC (defined as having two or more of the following symptoms, namely, the presence of a large fecal mass, feeling of obstruction when defecating, defecation disorder, laborious defecation, and defecation requiring hand assistance) for three months based on Rome IV diagnostic criteria. Notably, all included patients underwent both standardized DRE and HRAM test. Meanwhile, exclusion criteria were as follows: (i) patients who underwent abdominal operation and anorectal surgery, (ii) patients with potential secondary constipation causes, including organic lesions (such as colonic obstructions, intestinal tuberculosis, or colorectal cancer), drug-induced constipation (particularly opioids), neurological disorders such as Parkinson disease and metabolic problems (such as diabetes mellitus or hypothyroidism), and (iii) patients who are unable to understand the study protocol.

A total of 265 patients were eligible for the study, while twelve patients were excluded from the study due to incomplete data and thirty-five patients were excluded based on the exclusion criteria (13 patients underwent abdominal operation, 15 patients underwent anorectal surgery, 4 patients had IBD, and 3 patients were unable to complete HRAM). Finally, 218 FC patients, including 21 inpatients and 197 outpatients, who underwent a standardized DRE and HRAM, were enrolled and included in this study. The protocol used in the study was approved by the Ethics Committee of Anhui Provincial Hospital (Registration No.: 2016-L36). In addition, the protocol was registered on Chinese Clinical Trial Registry (No. ChiCTR-2100046993). All participants signed written informed consent before being included in the study.

#### 2.1.2. DRE

All included patients underwent a standardized DRE, and the results were recorded by a single expert investigator (Y Y). First, anocutaneous reflex and the surface of anus and surrounding tissue were thoroughly evaluated for any abnormalities. Second, the investigator conducted thorough digital palpation to assess patients' anorectal function. The anal resting pressure was determined and categorized as normal, decreased, or increased. Following that, patients were requested to squeeze for determining the anal squeeze pressure, which was categorized as normal, decreased, or increased. Third, the subject was requested to push and bear down as if to defecate. During this maneuver, anal sphincter relaxation, abdominal push effort, and perineal descent were assessed. Anal sphincter relaxation was categorized as normal, impaired relaxation, or paradoxical contraction, whereas the abdominal push effort was categorized as normal or impaired. Perineal descent was categorized as normal, absent, or excessive [7]. In this study, DRE report form and template were filled and recorded by the investigator (Y Y).

#### 2.1.3. HRAM

All participants underwent a HRAM test (Med Kinetic, Ningbo, China), and the results were recorded by a single experienced investigator (J L), as described in our previous research [13]. An anorectal catheter was inserted in each patient's rectum, and anal resting pressure (ARP) was measured using a water-perfused anorectal manometric catheter equipped with eight pressure sensors at 1 cm interval. The device utilizes a patented pressure transduction technology, allowing each pressure-sensor element to detect pressure over a length of 2.5 mm in each of the 12 radially dispersed sectors. After determining ARP, the anal squeeze pressure was measured as the highest squeeze pressure value obtained during five seconds of voluntary anal sphincter contraction. During simulated evacuation, rectal and anal pressures were measured before and after distension of a rectal balloon with 50 mL of water [14]. All data for each patient were analyzed using ManoView software.

#### 2.1.4. Data Analysis

Clinical information for each patient was collected, including age, gender, symptoms, and underlying diseases. DRE and HRAM were performed and the outcomes were recorded by different physicians (Y Y and J L), while DRE and HRAM results were analyzed by a third investigator (Y W) under blinded conditions. In this study, we explored the diagnostic yield of DRE, while HRAM was considered as a reference standard for DD diagnosis. The paradoxical anal contraction was defined as an increase in anal pressure compared with the resting status when the patients were requested to defecate [15, 16]. DD diagnosis by HRAM was based on the diagnostic criteria proposed by Soh et al. [8], while standardized DRE was proposed by Tantiphlachiva et al. [7].

### 2.2. Statistical Analysis

The agreement between DRE and HRAM was calculated using Cohen's kappa coefficient. In FC patients, the diagnosis value for DD (sensitivity, specificity, positive (PPV), and negative predictive values (NPV)) of DRE compared with HRAM and the agreement between the two tests were calculated. SPSS 21.0 software (SPSS Inc., Chicago, IL) was employed for statistical analysis. The difference was statistically significant when *P* < 0.05.

### 2.3. Results

#### 2.3.1. Demographics Data and Baseline Characteristics

A total of 265 patients were eligible for the study, while twelve patients were excluded from the study due to incomplete data and thirty-five patients were excluded based on the exclusion criteria. Finally, this study enrolled 218 FC patients, including 21 inpatients and 197 outpatients, with a median age of 59.08 ± 7.50 years (range: 16–85 years), and 96 (44.04%) were men. By DRE, 84 patients (38.53%) demonstrated inability to contract the abdominal muscles, whereas 72 patients (33.03%) demonstrated inability to relax the anal sphincter. The absence of perineal descent and paradoxical contraction was observed in 89 (40.83%) and 99 patients (45.41%). By HRAM, 101 patients (46.33%) had a dyssynergic pattern of defecation based on established criteria, [8] while 117 patients (53.67%) were diagnosed without DD. In detail, 39 patients had adequate rectal propulsion with paradoxical anal contraction, 23 patients had impaired rectal propulsion associated with paradoxical anal contraction, 28 patients had adequate rectal propulsion with an incomplete anal relaxation, and 11 patients had impaired rectal propulsion with incomplete anal relaxation.

#### 2.3.2. Diagnostic Accuracy of DRE for DD

By HRAM, a total of 101 patients (46.33%) were diagnosed with DD based on established criteria, [8] while 117 patients (53.67%) were diagnosed without DD. Meanwhile, a total of 72 patients (33.03%) were diagnosed with DD using DRE and HRAM, while 89 patients (40.83%) were classified as normal using both tests. A total of 28 patients were identified with dyssynergia using DRE, but they had normal coordination patterns when confirmed using HRAM. On the other hand, 29 patients were normal using DRE, whereas they were diagnosed with dyssynergia using HRAM. There was a moderate agreement between DRE and HRAM for diagnosing DD (*κ*-coefficient = 0.474, *P* < 0.001). The overall sensitivity of DRE in diagnosing dyssynergia was 71.3%, and the specificity was 76.1%. PPV was 72.0%, and NPV was 75.4% (Table 1).

## 3. Meta-Analysis

### 3.1. Search Strategy

In the current retrieval, English databases, including PubMed, Web of Science, Cochrane Library, and Embase database, were searched by combining subject terms and free words. The searching terms comprised dyssynergic defecation (constipation, chronic constipation, functional constipation, outlet obstruction constipation, defecatory disorders, dyssynergic defecation, dyssynergic evacuation, pelvic floor dysfunction, pelvic floor dyssynergia, and paradoxical contraction) and digital rectal examination (digital rectal examination, digital rectal exploration, digital anal rectal examination, digital anorectal examination, and digital rectal exam). By employing the abstraction in PubMed as an example, the concrete retrieval approaches constituted:

  #1 constipation [Mesh Terms]
  #2 dyssynergic defecation [Mesh Terms]
  #3 constipation [Title/Abstract]
  #4 chronic constipation [Title/Abstract]
  #5 functional constipation [Title/Abstract]
  #6 outlet obstruction constipation [Title/Abstract]
  #7 defecatory disorders [Title/Abstract]
  #8 dyssynergic defecation [Title/Abstract]
  #9 dyssynergic evacuation [Title/Abstract]
  #10 pelvic floor dysfunction [Title/Abstract]
  #11 pelvic floor dyssynergia [Title/Abstract]
  #12 paradoxical contraction [Title/Abstract]
  #13: #1 OR #2 OR #3 OR #4 OR #5 OR #6 OR #7 OR #8 OR #9 OR #10 OR #11 OR #12
  #14 digital rectal examination [Mesh Terms]
  #15 digital rectal examination [Title/Abstract]
  #16 digital rectal exploration [Title/Abstract]
  #17 digital anal rectal examination [Title/Abstract]
  #18 digital anorectal examination [Title/Abstract]
  #19 digital rectal exam [Title/Abstract]
  #20 #14 OR #15 OR #16 OR #17 OR #18 OR #19
  #21 #13 AND # 20.

The retrieval time of each database is from inception to June 1, 2021. Concurrently, the reference of related literature and reviews are retrieved manually to ensure that no omission occurs, and the studies in which constipated patients were evaluated using DRE are statistically analyzed. The protocol of this meta-analysis has been prospectively registered in PROSPERO (International Prospective Register of Systematic Reviews) database (No. CRD42021256572).

### 3.2. Study Selection

The studies that fulfill the following criteria were eligible for inclusion: (i) participants: constipated patients without a defined subtype of constipation and criteria for DD were clearly stated; (ii) intervention and comparison: patients were evaluated using DRE, and comparative physiological test was defined by the author; (iii) outcomes: sensitivity, specificity, positive predictive value (PPV), and negative predictive value (NPV) were reported in included studies, or the data were sufficient to determine true positives, false negatives, false positives, and true negatives of DRE for DD; (iv) study design: diagnostic studies (prospective or retrospective studies). Meanwhile, the exclusion criteria were as follows: (i) duplicate publications; (ii) studies without sufficient data; (iii) population with less than 20 patients.

### 3.3. Literature Quality Evaluation and Data Extraction

Two reviewers (JL and YW) independently screened literature according to the inclusion and exclusion criteria outlined previously. When disagreements arise, they consult and negotiate with a third participant (C S) to resolve the issue. The following data were extracted: first author's name, the time of publication, gender distribution, mean age, criteria for a constipation diagnosis, DRE procedures, criteria for positivity in DRE, comparative anorectal physiological test, criteria for diagnosing DD in physiological test, sensitivity, specificity, PPV and NPV of DRE for DD, or the data sufficient to determine number of true positive, true negative, false positive, and false negative. The validated Quality Assessment of Diagnostic Accuracy Studies 2 (QUADAS-2) tool was employed to assess the quality of included studies by two independent reviewers (JL and YW) [17, 18].

### 3.4. Statistical Analysis

This meta-analysis was conducted using a bivariate mixed-effects regression model [19]. Pooled estimates of sensitivity and specificity with corresponding 95% confidence intervals (CI) were combined using random-effects models. Heterogeneity was estimated using *I*^2^ statistic as well as *Q* statistic. Minimal heterogeneity was considered when *I*^2^ < 25%, while a cut-off of 50% represents moderate heterogeneity, and 75% represents high heterogeneity [20]. Pooled effects are presented in a forest plot with a corresponding 95% CI. A subgroup analysis of included studies was conducted based on different countries and different constipation criteria. Deek's funnel plot was assessed for publication bias. All analyses were performed using Stata 11.0 statistical software (Stata Corp., College Station, TX).

### 3.5. Results

#### 3.5.1. Eligible Studies

**Figure 1:**
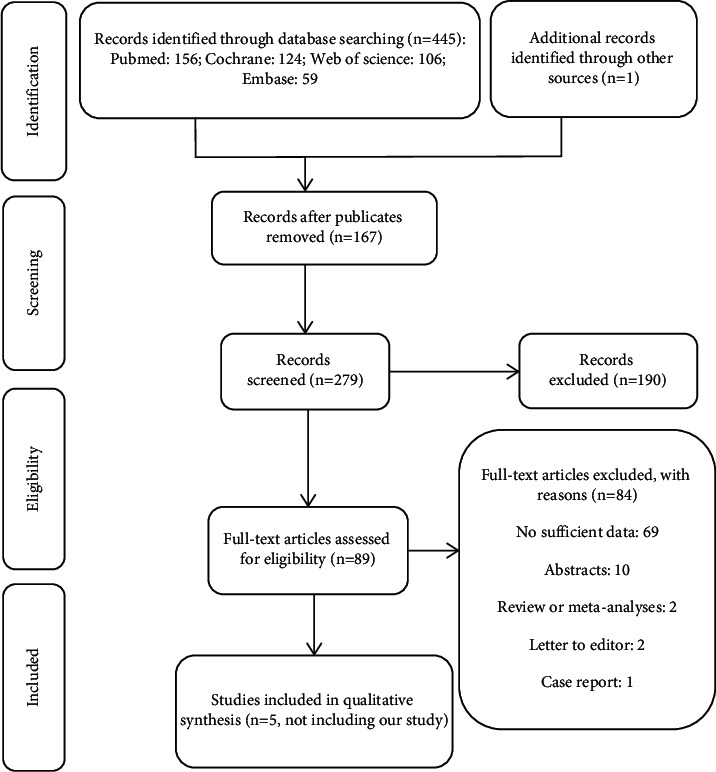
Flowchart of the literature search and study selection process.

We screened 279 titles and abstracts via electronic databases and manual search, and 89 full-text manuscripts were further reviewed. Finally, five articles fulfilled the criteria for inclusion and were included in this meta-analysis [7, 8, 21–23]. The graphic flow is depicted in Figure 1. The included articles were published from 1998 to 2018. The subjects of included studies were varied between 60 and 253 patients completing a total of 964 constipated patients (including our study). Rome criteria for constipation were used in two studies (not including our study) [7, 23]. The detailed information of included studies is displayed in Table 2.

#### 3.5.2. Quality and Risk of Bias Assessment

All five studies (not including our study) were considered a possible risk of bias according to QUADAS-2 criteria (Table 2). Regarding patient selection, two studies [7, 23] reported that included constipated patients fulfilled Rome criteria. However, two studies [7, 22] did not report the process and progress of DD diagnosis regarding flow and timing. Meanwhile, the reference standard of one study [23] was not determined using manometry (Table 3).

#### 3.5.3. Digital Rectal Examination

**Figure 2:**
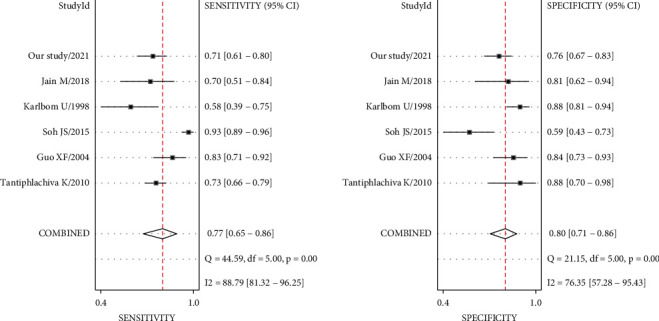
Pooled sensitivity and specificity of digital rectal examination for diagnosis of dyssynergic defecation (the outcome showed 77% summary sensitivity (95% CI: 65–86) and 80% summary specificity (95% CI: 71–86) to diagnose dyssynergic defecation).

**Figure 3:**
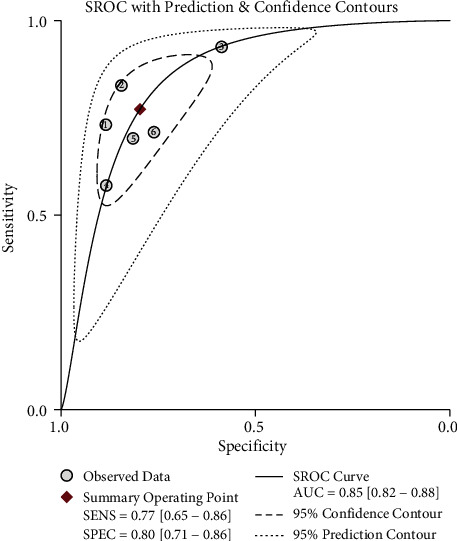
Summary receiver operator curve evaluating digital rectal examination as a diagnostic test for dyssynergic defecation (AUC was 0.85 (95% CI 0.82–0.88)).

**Figure 4:**
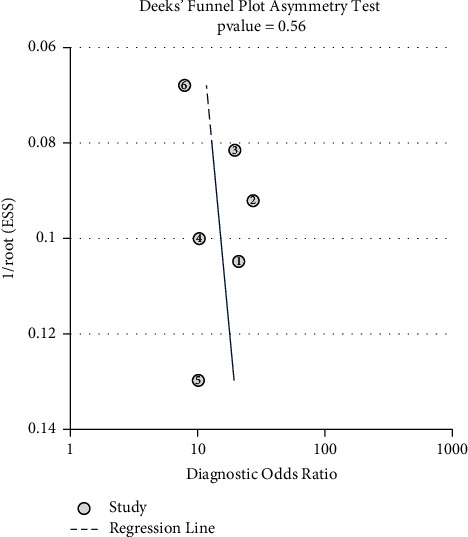
Publication bias evaluating digital rectal examination as a diagnostic test for dyssynergic defecation. There was no evidence of publication bias (*P*=0.56 > 0.05).

Six studies (including our study) considering DRE were included after careful evaluation. The meta-analysis demonstrated high heterogeneity between studies regarding summary sensitivity (*I*^2^ = 88.79%), while *I*^2^ = 76.35% regarding summary specificity (Figure 2). AUC was 0.85 (95% CI 0.82–0.88) with 77% summary sensitivity (95% CI 65–86) and 80% summary specificity (95% CI 71–86) to diagnose DD (Figure 3). There was no evidence of publication bias (*P*=0.56 > 0.05) (Figure 4).

#### 3.5.4. Subgroup Analysis

Subgroup analysis was performed according to different countries and different constipation criteria. For western countries, the pooled sensitivity (95% CI) for DD diagnosis with DRE using any criteria was 67% (CI 48–86%) and the pooled specificity was 88% (CI 80–95%), and no heterogeneity between studies was established by the meta-analysis (*I*^2^ = 0%). For Asian countries, the pooled sensitivity (95% CI) for DD diagnosis with DRE using any criteria was 82% (CI 72–91%), and the pooled specificity was 75% (CI 67–83%) and the moderate heterogeneity between studies was established by the meta-analysis (*I*^2^ = 42%). For constipation criteria of Rome, the pooled sensitivity (95% CI) for DD diagnosis with DRE using any criteria was 76% (CI 61–91%) and the pooled specificity was 81% (CI 70–91%), and no heterogeneity between studies was established by the meta-analysis (*I*^2^ = 0%). For studies without constipation criteria, the pooled sensitivity (95% CI) for DD diagnosis with DRE was 79% (CI: 65–93%) and the pooled specificity was 79% (CI: 67–90%), and no heterogeneity between studies was established by the meta-analysis (*I*^2^ = 0%). The subgroup analysis results indicated that heterogeneity of this meta-analysis might be due to different countries and different constipation criteria.

## 4. Discussion

In this study, we compared the diagnostic value of DRE to that of HRAM and discovered that DRE exhibits higher sensitivity and specificity, as well as a moderate agreement, in diagnosing DD than HRAM. As a result, DRE should be performed as a bedside screening test for DD diagnosis. Meanwhile, we conducted an updated meta-analysis based on the existing studies to further explore the pooled diagnostic accuracy of DRE for DD. This meta-analysis investigated the effectiveness of DRE for DD screening, and our results revealed that DRE was associated with an AUC of 0.85 (95% CI 0.82–0.88) with 71.3% summary sensitivity and 76.1% summary specificity in diagnosing DD.

Anorectal and pelvic floor disorders are relatively prevalent diseases, affecting 10–25% of the population [24, 25]. If patients had persistent or refractory symptoms of anorectal disorders, anorectal physiologic tests should be performed according to the latest guidelines [26, 27]. HRAM and balloon expulsion test (BET) are recommended as the first modalities to evaluate these patients. The principal objective of performing physiologic tests for constipated patients is to identify those with DD, as these patients would respond well to biofeedback therapy [13, 28]. In short, the results of anorectal physiologic tests were directly associated with subsequent treatment choices for constipated patients. Notably, due to the scarcity of HRAM, patients must be referred to tertiary centers with sufficient qualifications and expertise to conduct anorectal physiological evaluations. Although BET is a relatively simple test, the low sensitivity of BET in DD diagnosis was demonstrated in previous meta-analysis, rendering it unsuitable for use as a screening test, particularly when BET was performed in patients with left lateral position [29].

In clinical practice, DRE has been utilized as bedside physical examination for patients with anorectal symptoms. DRE can be easily conducted at the bedside and independent of any preparation or equipment, which becomes the important advantage of DRE. However, DRE is a technology that depends on operator's experience. To mitigate this bias, DRE was always executed by the same operator (Y Y) at our center. Moreover, DRE is just a screen tool and usually conducted at initial stages, allowing for early diagnosis. Therefore, if a positive DRE was found in patients, subsequent anorectal physiologic tests were required. The first study to estimate the accuracy of DRE compared with conventional anorectal manometry in dyssynergia diagnosis was performed by Tantiphlachiva et al. [7]. In that study, 209 patients with chronic constipation received standardized DRE and anorectal manometry. Compared with anorectal manometry, DRE demonstrated a high yield in screening DD with high specificity (87%) and PPV (97%); good sensitivity (75%) and a low NPV (37%). The latest study by Jain et al. [22] demonstrated that the detection rate of DRE for DD was 69.7% (23/33). The current meta-analysis indicates that DRE had an acceptable sensitivity and specificity compared with anorectal physiologic tests, implying that it can be considered a bedside tool for diagnosing DD in patients with functional constipation. One remarkable difference is that the specificity of DRE in this study was relatively lower than that in previous studies by Tantiphlachiva [7] and Jain [22] (>80% in both studies) compared with anorectal manometry. We postulated that this might result from the difference of measurement instruments compared with DRE because HRAM used more anal pressure sensors than conventional anorectal manometry. Another possible consideration was that most constipated patients referred to our center had persistent or refractory symptoms, implying that this study included fewer constipated patients with normal coordination patterns than that would be estimated in the general population. Therefore, more representative samples should be selected for future researches, and greater physiological details should be provided, which might improve DD evaluation [30].

Overdiagnosis for identifying DD might appear in DRE alone. In this study, the false positive was 28, indicating that 28 (12.84%) constipated patients were overdiagnosed using DRE alone for DD. It is reported that only 5 (8.33%) and 19 (7.51%) constipated patients were overdiagnosed DD in previous studies by Soh [8] and Jain [22], respectively. However, it should be emphasized that DRE is just a screening test with acceptable sensitivity for early diagnosis of DD before manometry but not a confirmatory test. Moreover, patients with positive DRE must be referred to tertiary centers with sufficient expertise to perform anorectal physiological evaluations. Most notably, if DD was identified, patients could follow biofeedback therapy in tertiary centers. In a recent meta-analysis, dyssynergia resolved in 72% (91/126) of patients treated with biofeedback therapy, and biofeedback therapy was superior to non-biofeedback therapy for resolution of dyssynergia (OR 9.43, CI 0.8–111.2, *Z* = 1.78, *P* < 0.00001, I^2^ = 93%).

This updated systematic review investigated the effectiveness of DRE for DD screening and pooled sensitivity and specificity of DRE for diagnosis of DD. Regarding patient selection, two studies [7, 23] stated that enrolled constipated patients fulfilled Rome criteria. Nevertheless, two studies [7, 22] did not report the process and progress of DD diagnosis regarding flow and timing. Meanwhile, the reference standard of one study [23] was not performed by manometry, which is considered the most important standard for DD. The heterogeneity in our meta-analyses was high (88.79% and 76.35% for sensitivity and specificity, resp.), which could be explained by the disparate definitions employed for abnormal DRE or by disparities in investigator training for performing DRE. Likewise, the reference standard tests were not identical. Notably, the heterogeneity of this meta-analysis may result from different countries and different constipation criteria across included studies based on subgroup analysis.

However, previous studies indicated that performing DRE has decreased clinical practice, and efforts to educate medical students about this modality are insufficient [11, 31]. Lawrentschuk et al. [11] reported that only 11% of students had palpated patients with constipation, and up to 17% did not perform it during medical school. Regarding perceptions and practice patterns of DRE, the results of a study conducted by Wong et al. [31] revealed that the diagnostic yield difference between well-trained gastroenterologists and less-trained medical students and physicians was not obviously significant in recognizing conditions such as bleeding per rectum or prostate enlargement using DRE. Notably, well-trained gastroenterologists had higher diagnostic accuracy in detecting dyssynergia and anal sphincter weakness using DRE than others. Consequently, adequate training and meticulous DRE application in clinics and DRE can provide useful clinical information about patients with anorectal disorders. Our current data highlight the importance of appropriate DRE training and performance.

### 4.1. Limitations

Our study has the following limitations. First, DRE is an operator-dependent technique, and one of the main deficits is that DRE was performed by one experienced clinician (Y Y). Therefore, it is unclear how well it would perform when done by another physician with less experience with DRE for the diagnosis of DD. Second, since most constipated patients referred from a clinic to our tertiary center had persistent or refractory symptoms, this study included fewer constipated patients with normal coordination patterns, resulting in a higher prevalence of dyssynergia in our study than in the general population. Third, the criteria to define constipation varied across included studies, ranging from not defined or self-reported to Rome criteria. Due to the lack of standardization in DRE, it was challenging to compare studies. Also, the absence of an actual comparative gold-standard test or unique diagnostic criteria for DD makes any review on the subject a difficult task. Finally, although rigorous exclusion criteria were used for enrolling patients, one of the confounding factors was that the results of inpatients were included and analyzed in the current study, which may have introduced bias. However, it is difficult to conduct a subgroup analysis because of low sample size of inpatients in our study and unknown numbers of inpatients mentioned in other studies.

## 5. Conclusion

In summary, this analysis results indicated that DRE could be a valuable and straightforward bedside tool for screening DD. Our diagnostic study and meta-analysis revealed acceptable sensitivity and specificity of DRE in detecting dyssynergia compared with physiological tests. Meanwhile, our study highlights that DRE remains an important tool in clinical practice. We recommend that each medical personnel should utilize DRE as a routine examination when evaluating patients with anorectal disorders.

## Figures and Tables

**Table 1:** Comparison of findings on digital rectal examination with high-resolution anorectal manometry.

	HRAM+	HRAM-	Outcomes
DRE+	72	28	Sensitivity 71.3%
DRE-	29	89	Specificity 76.1%
Total no. of cases	101	117	PPV 72.0%
	Patients diagnosed with DD using HRAM	Patients diagnosed without DD using HRAM	NPV 75.4%
			Detection rate 71.3% (72/101)
			Cohen kappa correlation coefficient = 0.474, *P* < 0.001

DRE: digital rectal examination; DD: dyssynergic defecation; HRAM: high-resolution anorectal manometry; PPV: positive predictive value; NPV: negative predictive value.

**Table 2:** General characteristics of the included studies.

Study reference	Country	Criteria of constipation	*N*	DRE	Comparative test	Extracted data
Soh et al. (2015)	Korea	NA	253	2 of as-pc/as-nr, pe-i, pd-a	HRAM (type I–IV DD)	tp = 193, fp = 19, fn = 14, tn = 27
Tantiphlachiva et al. (2010)	America	Rome III criteria	209	2 of as-pc/as-nr, am-nc, pd-a	AM	sensitivity = 75%, specificity = 87%, PPV = 97%, and NPV = 37%
Karlbom et al. (1998)	Sweden	NA	106	pr-pc	AM + CTT	tp = 19, fp = 12, fn = 14, tn = 91
Guo et al. (2004)	China	Rome II criteria	118	pr-pc	DEF + EMG	sensitivity = 82.53%, specificity = 85.21%,
						False positive rate = 14.82%,
						False negative rate = 17.52%
Jain et al. (2018)	India	NA	60	as-pc, as-nr, am-nc, pd-a	AM	tp = 23, fp = 5, fn = 10, tn = 22

DRE: digital rectal examination; HRAM: high-resolution anorectal manometry; AM: anorectal manometry; DEF: defecography; EMG: electromyography; CCT: colonic transit time; as-pc: anal sphincter paradoxical contraction; as-nr: anal sphincter nonrelaxing; am-nc: abdominal muscles not contracted; pd-a: perineal descent absent; pe-i: push effort impaired; pr-pc: puborectalis paradoxical contraction; PPV: positive predictive value; NPV: negative predictive value; tp: true positive; fp: false positive; fn: false negative; tn: true negative; NA: not available.

**Table 3:** Study quality assessment according to QUADAS-2 tool.

Study and year	Risk of bias	Applicability concerns					
	Patient selection	Index test	Reference standard	Flow and timing	Patient selection	Index test	Reference standard
Soh et al. (2015)	Unclear	Low	Low	Low	Low	Low	Low
Tantiphlachiva et al. (2010)	Low	Low	Low	High	Low	Low	Low
Karlbom et al. (1998)	Unclear	Low	Unclear	Unclear	Low	Low	Low
Guo et al. (2004)	Low	Unclear	Unclear	Low	Low	Low	Unclear
Jain et al. (2018)	Unclear	Low	Low	High	Low	Low	Low
Our study (2021)	Low	Low	Low	Low	Low	Low	Low

## Data Availability

The data used to support the findings of this study are available from the corresponding author upon request. Data were collected by authorized researchers.
